# Do supportive family behaviors promote diabetes self-management in resource limited urban settings? A cross sectional study

**DOI:** 10.1186/s12889-018-5766-1

**Published:** 2018-07-04

**Authors:** Saranya Ravi, Swetha Kumar, Vijayaprasad Gopichandran

**Affiliations:** 1 ESIC Medical College & PGIMSR, KK Nagar, Chennai, 600078 India; 2 Department of Community Medicine, ESIC Medical College & PGIMSR, KK Nagar, Chennai, 600078 India

## Abstract

**Background:**

Self-management is an essential component of prevention and treatment of type 2 diabetes. Social and family support has been shown to influence self-management behaviors as well as glycemic control and complications. This study was conducted to assess whether diabetes family support improves diabetes self-management and glycemic control in a typical urban population in India.

**Methods:**

A cross-sectional study using a questionnaire that had items from the Summary of Diabetes Self Care Activities Scale (SDSCA), the Diabetes Family Behavior Checklist (DFBC) and some sociodemographic and diabetes related clinical data was conducted. The participants were consecutively sampled from the diabetes outpatient department in a tertiary care hospital in Chennai, south India.

**Results:**

A total of 200 consecutive patients from the diabetes outpatient department were interviewed. Diabetes self-management practices were good with respect to avoiding fatty foods and carbohydrates and undergoing regular blood testing for glucose. But the self-management with respect to exercise and foot related care was rare. It was observed that a vast majority of the patients did not report receiving any support from their families. However, in the small proportion who did receive good family support, there is an association between diabetes self-management and diabetes family support (β = 0.527; *p* = 0.015). Further, the path model showed that there is a positive statistically significant association between family support score and the diabetes self-management score (β = 0.254, *p* < 0.001). However, the negative association between the diabetes self-management score and the mean plasma glucose did not reach statistical significance (β = − 46.378, *p* = 0.082).

**Conclusions:**

In the urban south Indian setting, family support was significantly associated with better self-management activities, but better self-management did not reflect as better glycaemic control.

**Electronic supplementary material:**

The online version of this article (10.1186/s12889-018-5766-1) contains supplementary material, which is available to authorized users.

## Background

India has a huge burden of type 2 diabetes. More than 1 million deaths are attributable to type 2 diabetes annually in India [1]. A major part of treatment of type 2 diabetes is life style modification. Therefore, the treatment focuses on imparting diabetes self-management education (DSME) to the patients. The main aspects of self-management include healthy eating, being active, monitoring blood glucose levels, regular intake of medicines, problem solving, risk reduction and healthy coping [2]. There is enough evidence to show that good self-management behaviors lead to better glycemic control [2–4]. Intensive self-management has been shown to reduce diabetes complications such as nephropathy, stroke, cardiovascular complications, and foot amputations [5, 6]. When adopted by healthy persons, self-management has also been shown to prevent type 2 diabetes [7].

Several key factors influence the adoption and maintenance of self-management of diabetes. The most important among these are personality characteristics of the patient with diabetes, characteristics of the health care provider, the provider-patient relationship and support received from the community [8]. Family is an integral provider of support for patients with diabetes. Families provide physical support to the patient, help in maintaining a structure and organization for effective life style adoption, and also provide emotional support at times of need [8].

India, a previously agrarian society, is rapidly urbanizing. The characteristic features of an urban family setting are nuclear family, individualistic tendencies of the family members, lack of cohesion within the families and unfavorable economic circumstances [9]. In such a family setting, it is important to note the level of family support that is available for patients with diabetes. It is also important to understand how this family support influences the self-management practices of the patients. In a dominantly patriarchal society, it is likely that family dynamics may adversely affect the women from practicing self-management behaviors, whereas it may be favorable for men [10]. While families may buffer the psycho-social stresses that a patient with diabetes is subject to, it may also act as the primary cause for these stresses [11]. This cross sectional study was conducted in Chennai, south India to understand the levels of family support to patients with diabetes and to assess the influence of family support on self-management of patients with diabetes and their glycemic control.

## Methods

This cross-sectional study was conducted in Employees State Insurance Corporation (ESIC) Hospital, a tertiary care hospital in Chennai, south India. The patients were employees earning less than Rs. 21,000 (USD 320) per month and their family members, who were insured under the social insurance scheme of the Ministry of Labor and Employment, Government of India. The patients attending the diabetic outpatient department were included in the study. Usually, the patients with diabetes, who are under fair control of blood sugars, visit the hospital once in 3 months to check their fasting and post-prandial blood glucose levels and consult the diabetologist to get their medication refill. In a previous study the prevalence of good self-management among patients with diabetes was found to be 45% [12]. Hence a sample size of 200 was calculated to estimate the association between family support and good self-management practices for a 95% confidence level and 80% power.

Adult men and women who were diagnosed to have type 2 diabetes mellitus were included in the study. The study was conducted on patients attending the outpatient department from May 2017 to September 2017. Patients who were dependent on others for their activities of daily living were not included as their self-management practices cannot be assessed. Patients who had cognitive impairment and any disability in the nervous system were also excluded from the study as the self-management activities of these patients will be affected.

The researcher asked questions to the patients from the questionnaire which included two domains - self management behaviors and family support in addition to general socio-demographic characteristics. The questionnaire to assess self-management was adopted from the Summary of Diabetes Self Care Activities [13]. This included patients’ adherence to a proper diet pattern, avoidance of fatty foods, adequate fruit and vegetable intake, exercise plan, glucose monitoring and foot care. The patients were asked in how many of the past seven days they followed the self-management behaviors. The score was marked on a scale of 0 to 7 representing the number of days the behavior was followed. The Diabetes Family Behavior Checklist was used to assess the family support of the patient [14]. The questionnaire contained 16 questions. The questions were oriented on the family members’ support to the patient following his/her self-management behavior. This included appreciating the patients when they follow their diet pattern, reminding the patient to check their blood glucose levels, nagging the patient to follow their diet regularly, criticizing the patient for not following their exercise schedule, accompanying the patient during exercise, eating meals at the same time as the patient, and other such questions. The questions were scored on a scale of 1 to 5. Here 1 represented no support provided at all and 5 represented the patient is supported always. The questionnaires was translated in Tamil, the local language and used. The fasting and post prandial blood glucose levels of the patient were also recorded to understand their glycemic control levels. A written informed consent was obtained from all the patients prior to the study, informing the patient about the goals and procedures of the research. Ethical approval was obtained from the Institution Ethics committee, ESIC Medical College and PGIMSR, Chennai.

The collected data was entered in the computer using Microsoft Excel spreadsheet. The data was analyzed using IBM SPSS statistical software version 21 to find the relation between family support and self-management behaviors of the patient. The median number of days during which the self-management activity was carried out in the past 1 week was computed. The number of days were also treated as the individual patients’ respective scores on the SDSCA scale and the scores were summed-up. The total score for each patient was calculated as the self-management score. The frequency of support in each dimension was evaluated as a median frequency using the diabetes family behavior checklist. The frequency of family support was also converted to a numerical score and added to yield the family behavior support score. Two main hypotheses were tested using structural equation modeling and path analysis. The first was to evaluate the two latent variables namely, diabetes self-management, diabetes related family support and study the association between the two. The second analysis was to establish a path model to study relationship between family support, diabetes self-management and glycemic control, after adjusting for influences of age, sex, type of family, education and monthly family income. The structural equation modeling analysis was performed using IBM AMOS software version 21. The structural model comprised of two latent variables namely, diabetes family behavior support and diabetes self-management activities. Each item in these scales were loaded as the respective observed variables. Model fit indices such as Comparative Fit Index (CFI), Normed Fit Index (NFI) and Root Mean Square Error Approximation (RMSEA) were assessed. Acceptability of the model fit were tested based on these indices [15]. A path model was also constructed using the total diabetes family support score, the diabetes self-management score and the mean plasma glucose. Age, sex, family type, education and monthly family income of the participants were entered as covariates in the model.

## Results

A total of 200 patients in the diabetic outpatient department were approached for the study and all of them consented to participate and responded to the questions. The characteristics of the study sample are shown in Table 1. There were almost an equal distribution of men and women. About 85% of the study sample were between the ages of 41 and 70 years. Only 38% had education above high school level. More than 75% earned less than Rs. 12,000 per month (USD 180). About 57% had diabetes for 5 years of lesser. A majority of 91% of the participants came from nuclear families.

**Table 1:** Characteristics of the study population

Characters	Categories	frequency	Percentage
Sex	Male	96	48
	Female	104	52
Age	31–40	20	10.1
	41–50	60	30.2
	51–60	55	27.6
	61–70	54	27.1
	71–80	9	4.5
	81–90	1	0.5
Education	Uneducated	46	23.1
	Primary School	41	20.6
	Middle school	37	18.6
	High school	69	34.7
	UG and PG	6	3
Family income per month	< 1600	5	2.8
	1601–4800	19	10.6
	4801–8009	52	29.1
	8010–12,019	62	34.6
	12,020–16,019	24	13.4
	> 16,020	17	9.5
Diabetic since	less than 5 years	105	56.8
	5–10 years	40	21.6
	> 10 years	40	21.6
Type of family	Nuclear	182	91
	Joint	18	9

### Diabetes self-management behaviors

Table 2 depicts the self-management behavior of the patients. It is represented as the median number of days in the past week that the sample of participants followed the self-management behavior. It is seen that while diet and blood test related self-management was good, the behaviors related to exercise and foot care were very poor.

**Table 2:** Summary Diabetes Self Care Activities (SDSCA)

Question	Median Number of days in the past week	IQR
How many of the last SEVEN DAYS have you followed a healthful eating plan?	6	2–6
On average, over the past month, how many DAYS PER WEEK have you followed your eating plan	6	2–6
On of the last SEVEN DAYS did you eat five or more servings of fruits and vegetables?	0	0
On how many of the last SEVEN DAYS did you avoid high fat foods such as red meat or full-fat dairy products?	6	5–7
On how many of the last SEVEN DAYS did you participate in at least 30 min of physical activity?	0	0–7
On how many of the last SEVEN DAYS did you participate in a specific exercise session (such as swimming, walking, biking) other than what you do around the house or as part of your work?	0	0–7
On how many of the last THREE MONTHS did you test blood sugar?^a^	1	0–1
On how many of the last THREE MONTHS did you test your blood sugar the number of times recommended by your health care provider?^a^	1	0–1
On how many of the last SEVEN DAYS did you check your feet?	0	0
On how many of the last SEVEN DAYS did you inspect the inside of your shoes?	0	0
Did you smoke even a puff of cigarette in the past 7 days?	0	0

^a^in the study setting blood test for sugar level is advised only once in 3 months. Therefore, this indicates frequency in the past 3 months

### Family support for diabetes

The Diabetes Family Behavior checklist exhibited an excellent internal consistency with a Cronbach’s Alpha of 0.896. It is seen in Table 3 that the diabetes related family support is very poor in the sample that was studied. The median response for most of the supportive and non-supportive family behaviors was only ‘never’. The instances of having a frequent support was limited to suggesting ideas to take medicines regularly, nag and argue with them to adopt healthy life styles, and eat at the same time as the patient. Further it was seen that none of the sociodemographic covariates had a statistically significant influence on the family support. This analysis is shown in Additional file 1: Table S1.

**Table 3:** Diabetes Family Behavior Checklist

Questions	Median Score (frequency in the past month)	IQR
Praise the patient for following his/her diet	1	1–3
Nag him/her about testing their blood glucose level	1	1–3
Suggest things that may help him/her take their medicines on time	1	1–5
Criticize them for not exercising regularly	1	1–3
Help them decide of any lifestyle changes need to be made based on glucose testing results	1	1–5
Nag them about following their diet	1	1–5
Argue with them about their diabetes self-care activities	1	1–5
Encourage them to participate in sports / physically active leisure time activities	1	1–3
Plan family activities so that it will fit in with their diabetes self-care schedule	1	1–1
Congratulate them for sticking to their diabetes self-care schedule	1	1–1
Criticize them for not maintaining the results of glucose test	1	1–4
Eat at the same time that they do	1	1–5
Exercise along with them	1	1–1
Let them sleep longer and get up later rather than exercise	1	1–1
Buy them sweet things that they can keep with them in case of low blood sugar conditions	1	1–1
Eat foods that are not part of their diabetic diet	1	1–3

1 – never, 2 – twice a month, 3 – once a week, 4 – several times a week, 5 – at least once a day

### Structural equation model of relationship between diabetes family support and self-management

**Fig. 1:**
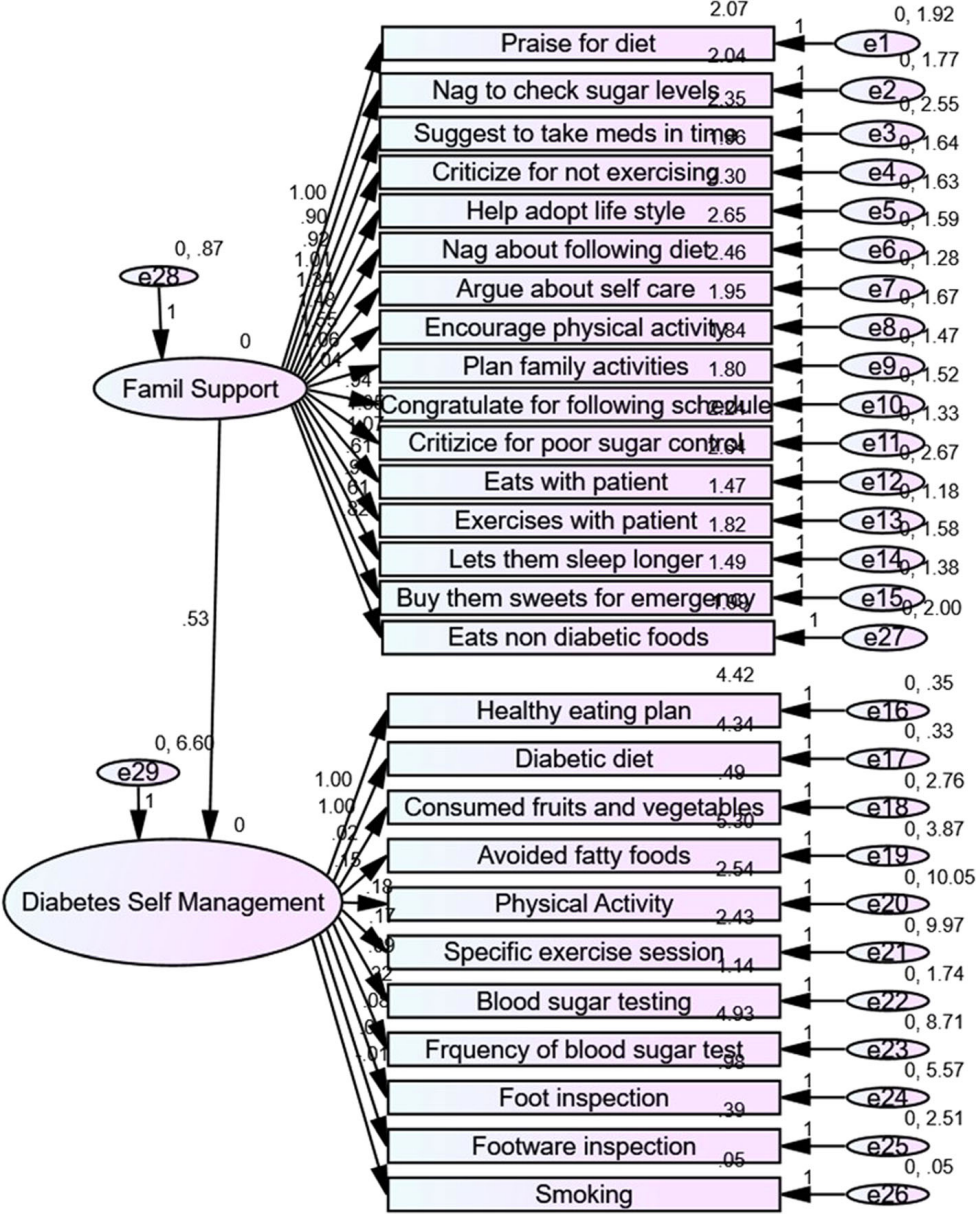
Structure Equation Model of relationship between family support and diabetes self-care activities. This figure shows the structural equation model of association between family behaviour checklist and the diabetes self-management. The model shows the relationship between the various items of each scale with the latent variable as well as the association between the two latent variables. The model has acceptable fit as indicated by CFI = 0.925 (greater than 0.90 indicates acceptable fit), NFI = 0.899 (greater than 0.80 indicates acceptable fit), RMSEA = 0.076 (lesser than 0.08 indicates acceptable fit)

The structural equation model had acceptable model fit, as indicated by Comparative Fit Index = 0.925 (greater than 0.90 indicates acceptable fit), Normed Fit Index = 0.899 (greater than 0.80 indicates acceptable fit), Root Mean Square Error Approximation = 0.076 (lesser than 0.80 indicates acceptable fit). It is seen in Fig. 1 as well as Table 4 that all the questions in the diabetes family behaviour checklist loaded in a statistically significant manner on to the latent variable. With respect to diabetes self-management behaviours, intake of fruits and vegetables, foot care, care of the footwear and smoking did not load significantly on to the latent variable of self-management. All other questions loaded in a statistically significant manner. Further it can be seen that there is a statistically significant positive association between family supportive behaviour and diabetes self-management (β = 0.53; *p* = 0.015).

**Table 4:** Association between Family Behavioral Support and Summary Diabetes Self Care Activities

Dependent Variable	Independent Variable	Regression Weight	S.E.	P
SDSCA	DFBC	.53	.216	.015
Praise for following diet	DFBC	1.00		
Nag to check sugar levels	DFBC	.90	.146	< 0.001
Suggestions to take medicines in time	DFBC	.92	.163	< 0.001
Criticize for not exercising	DFBC	1.00	.152	< 0.001
Help adopt healthy lifestyle	DFBC	1.33	.180	< 0.001
Nag about following healthy diet	DFBC	1.48	.194	< 0.001
Argue about selfcare	DFBC	1.55	.195	< 0.001
Encourage physical activity	DFBC	1.06	.157	< 0.001
Plan family activities together	DFBC	1.04	.151	< 0.001
Congratulate for following exercise schedule	DFBC	.94	.143	< 0.001
Criticize for poor blood sugar control	DFBC	1.34	.176	< 0.001
Eats with the patient	DFBC	1.07	.177	< 0.001
Exercises with the patient	DFBC	.60	.110	< 0.001
Lets them sleep longer and compromise exercise	DFBC	.96	.147	< 0.001
Buys them sweets to overcome emergencies	DFBC	.60	.116	< 0.001
Eats non-diabetic foot for taste	DFBC	.82	.145	< 0.001
Healthy eating plan	SDSCA	1.00		
Healthy eating behavior	SDSCA	.99	.051	< 0.001
Consumes fruits and vegetables	SDSCA	.02	.046	.649
Avoids fatty food	SDSCA	.14	.054	.007
Involves in physical activity	SDSCA	.18	.087	.038
Does regular recommended exercise	SDSCA	.17	.087	.044
Tests blood sugar parodically	SDSCA	.08	.036	.019
Blood tests done as advised by doctor	SDSCA	.31	.081	< 0.001
Inspection of the feet	SDSCA	.08	.065	.199
Inspection of inner aspects of shoes	SDSCA	.01	.044	.843
Smokes even 1 cigarette / other tobacco	SDSCA	−.01	.007	.164

*SDSCA* Summary Diabetes Self Care Activities, *DFBC* Diabetes Family Behavior Checklist

### Association between diabetes family support, self-management and mean plasma glucose

**Fig. 2:**
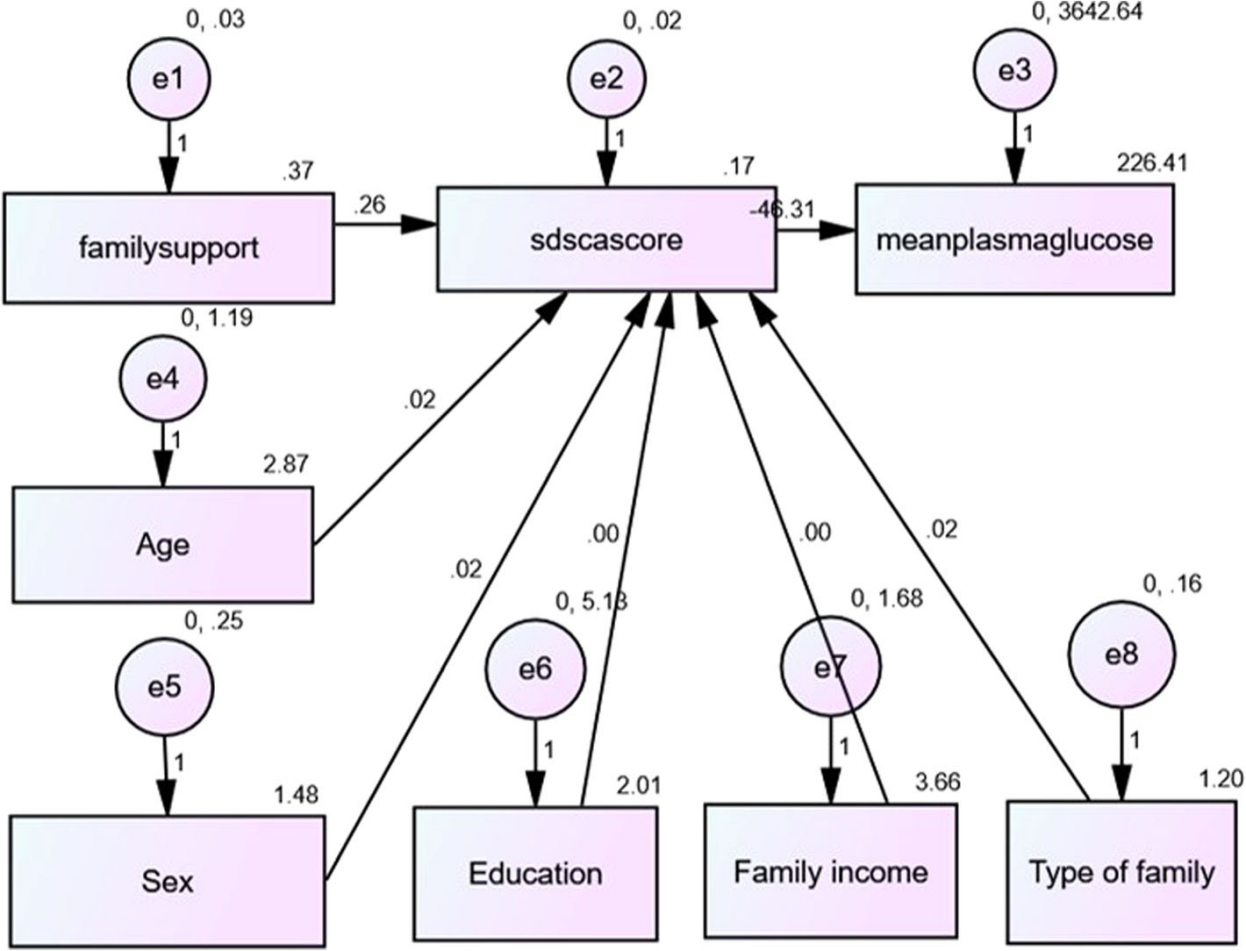
Path model to assess relationship between family support, diabetes self-management and mean plasma glucose. This path model shows the regression coefficients of association between family support, diabetes self-management and mean plasma glucose. It is seen that there is a positive association between family support and diabetes self-management, whereas there is a negative association between self-management and mean plasma glucose. However, the association between self-management and mean plasma glucose was not statistically significant. Age had a statistically significant influence on the self-care activities, whereas sex did not have an influence. The model has acceptable fit as indicated by CFI = 0.990 (greater than 0.90 indicates acceptable fit), NFI = 0.956 (greater than 0.80 indicates acceptable fit), RMSEA = 0.042 (lesser than 0.08 indicates acceptable fit)

A path model was constructed between total score of family support, total score of self-management and the mean plasma glucose which is shown in Fig. 2. In this model, age, sex, family type, education and monthly family income of the participants were entered as covariates. It is seen that there is a positive statistically significant association between family support score and the diabetes self-management score (β = 0.26, *p* < 0.001). However, the negative association between the diabetes self-management score and the mean plasma glucose did not reach statistical significance (β = − 46.31, *p* = 0.082). Further, the model showed that age of the participant had a positive correlation with self-management scores (β = 0.02; *p* = 0.026) but sex of the participant did not have a statistically significant association (β = 0.02; *p* = 0.405). The other variables such as education, family type and monthly family income also did not have an influence.

## Discussion

This study explored the association between diabetes family support, diabetes self-management behaviors and glycemic control in patients with diabetes attending a tertiary care hospital in south India. The typical finding was almost a complete lack of any form of supportive family behavior in the study sample with very few people reporting any form of family support. The self-management behaviors were good in diet and blood testing, but very poor in exercise and foot care. It was found that the few people who had greater family support for diabetes had better self-management behaviors. There was also a negative association observed between self-management behaviors and glycemic control as measured by mean plasma glucose, but this association was not statistically significant.

### Poor diabetes self-management behaviors

Several previous studies from India have reported poor diabetes self-management behaviors. As seen in previous studies, the self-management behaviors related to diet and blood testing were good in this study sample as well [12, 16]. While diet practices have consistently been observed to be good in the patients with diabetes in India, exercise and foot care have been neglected. This highlights the importance of emphasis on exercise and foot care in the diabetes self-management education programs in India. Even in the diet practices, the behaviors that were more commonly observed were reduction in intake of carbohydrates, sugars and fats. However, consumption of fruits and vegetables was very low. This was also seen in the structural equation model, in which fruits and vegetable consumption do not feature as a significant independent variable contributing to the latent variable of diabetes self-management. The reason for this is the prohibitive cost of fresh fruits and vegetables in the local area. Diabetes self-management support and education should also emphasize on household gardens and local cultivation of vegetables which can be used frequently in cooking. The diabetes self-management education should also focus on enabling policy environment where the produce and sale of vegetables and fruits should be subsidized by the government.

### Poor diabetes related family support

Though the Diabetes Family Behavior Checklist that was used in this study originally comprises of two subscales – supportive behaviors and non-supportive behaviors, in this study the scale performed as a unidimensional scale as seen in the structural equation model [17]. The supportive behaviors in the original scale comprised of items that emphasized on appreciating the patient for practicing a certain behavior. On the other hand there were the non-supportive behavior items where the statements pertained to nagging or criticizing the patient for not following certain self-management behaviors [18]. It was reported in studies conducted in the West that, while the supportive behaviors improved diabetes self-management, the non-supportive ones hampered it [19, 20]. However, when the scale was used in the Indian context in this study, it performed as a unidimensional one, which means that both the appreciation for following a certain behavior and criticism and nagging for not following a behavior, were viewed as forms of supportive behavior. Both these behaviors were seen as the family member being involved in the care of the patient with diabetes, the opposite of this being apathy towards the patient’s self-management. It was also seen that this family support was overall very poor. Among those family supportive behaviors, the ones that were more common were diet related and overall self-management advice related.

Several social structural issues are the basis for the poor diabetes related family support observed in the typical urban south Indian population sampled in this study. Firstly, the sample was predominantly urban, with a fast and industrialized life. More than 90% were nuclear families. The number of members available to provide support to the diabetic patients is lesser in nuclear families compared to joint ones. The proportion of families which are nuclear in India according to census of 2011 was 70%. The study sample had a much greater proportion of nuclear families. Therefore, the findings of the study may be over-estimating the lack of family support, which needs to be borne in mind while interpreting the findings. Majority of the study sample were in the age group of 50 years and above. This also meant that some of them were retired, home-bound, not economically productive, living alone without the support of their sons and daughters and having nobody to care for or support them. Though India is a young country today, the numbers of elderly are fast increasing, and the demographic patterns of the elderly are dominated by loneliness and lack of support.

### Association between diabetes related family support and diabetes self-management

Studies have shown that diabetes self-management is dependent on four major factors namely, the characteristics of the patient, stress related to diabetes, characteristics of the diabetes care provider and the provider-patient relationship and finally characteristic of social support [8, 21]. Family support is a very important component of social support for diabetes. Several important characteristic features of families have been shown to be associated with poor diabetes self-management behaviors. These are, low family cohesion, high family conflict, too tight or too permeable family boundaries, low levels of organization of families and distant families [22]. It is increasingly seen that urban families, nuclear families and families in low and middle income settings with high levels of resource deprivation have a tendency to have these characteristic features [23]. This was observed in this study.

It was also seen that there was a statistically significant association between family support and diabetes self-management behaviors in the patients. This is consistent with several previous studies which have documented this both in observational as well as experimental settings [18, 24–30]. The structural equation model revealed that the construct of diabetes self-management was positively associated with the construct of diabetes related family support with a strong regression coefficient. This has important implications. Improving family relationships, increasing the involvement of families in care and support of diabetes is likely to improve diabetes self-management practices, in a typical urban Indian context as well. While the previous Western studies have shown that family behaviors can influence diabetes self-management based on whether they are supportive or non-supportive, in this study it is seen that any form of family engagement, supportive and appreciative or non-supportive and critical may lead to improved self-management.

### Failure to demonstrate association between family support, self-management and glycemic control

The path model revealed that while there is a statistically significant association between diabetes family support and diabetes self-management, there was no association between diabetes self-management and mean plasma glucose. The mean plasma glucose was computed by averaging the fasting and post-prandial glucose values of each patient [31]. Glycemic control was measured by measuring mean plasma glucose which is more likely to be variable than glycosylated hemoglobin. Probably measurement of glycosylated hemoglobin as done in most previous studies, would have established an association.

### Strengths and limitations of this study

To the best knowledge of the authors, this is the first study to explore family dynamics and support and its influence on diabetes self-management in an Indian context. The dynamics of Indian family structure is fast changing in a rapidly urbanizing India, and this is reflected in this study. It has important implications for planning diabetes self-management education in the country. The main limitation of this study is that it is hospital based and done only in a predominantly urban setting. Large part of the Indian population is rural and future studies should focus on rural community based studies. The other limitation is inability to measure glycosylated hemoglobin due to resource constraints. Future studies should also target developing unique family support scales for the Indian context, as the supportive/non-supportive paradigm of thinking seems to work differently in this context.

## Conclusions

This study shows that diabetes self-management behaviors can be substantially improved by improving family support. In this typical urban Indian context, family support was observed to be any form of engagement of the family members in the care of diabetes, irrespective of whether it was supportive or non-supportive. There is also an indication that increased family support may improve self-management which in turn may improve glycemic control. Therefore, future diabetes self-management education in India should focus on family engagement in care.

## Additional file

Additional file 1: **Table S1** Association between socio-demographic factors and family support. (DOCX 14 kb)

## Abbreviations

CFI: Comparative fit index
DFBC: Diabetes Family Behaviour Checklist
DSMB: Diabetes Self Management Behaviour
DSME: Diabetes Self Management Education
ESIC: Employees State Insurance Corporation
NFI: Normed fit index
RMSEA: Root Mean Squared Error Approximation
SDSCA: Summary of Diabetes Self Care Activities
