# Supplementary material for: Do supportive family behaviors promote diabetes self-management in resource limited urban settings? A cross sectional study
Source: BMC Public Health. 2018 Jul 4;18:826. doi: 10.1186/s12889-018-5766-1 (PMC6031108; doi:10.1186/s12889-018-5766-1)
Supplement: Supplementary file 1 — Table S1 Association between socio-demographic factors and family support. (DOCX 14 kb) [file 12889_2018_5766_MOESM1_ESM.docx]

Additional File 1

Table S1 Association between socio-demographic factors and family support

| S. No. | Socio-demographic covariate ^*^ | Adjusted Odds Ratio | 95% CI | p value |
| --- | --- | --- | --- | --- |
| 1 | Age | 0.784 | 0.536 – 1.148 | 0.212 |
| 2 | Sex | 2.213 | 0.809 – 6.054 | 0.122 |
| 3 | Occupation | 0.695 | 0.676 – 1.298 | 0.695 |
| 4 | Education | 0.970 | 0.848 – 1.187 | 0.970 |
| 5 | Monthly family income | 1.035 | 0.750 – 1.427 | 0.835 |
| 6 | Duration of diabetes | 1.148 | 0.686 – 1.921 | 0.600 |

*Family support score was computed and categorized into good and low support using the mean score for this analysis. Multiple logistic regression analysis was performed.
